# Extent of cardiovascular medications dispensing practice without a prescription: Self-reported and simulated patient-based study at community pharmacies in Northwest Ethiopia

**DOI:** 10.1186/s40545-023-00533-4

**Published:** 2023-02-24

**Authors:** Ashenafi Kibret Sendekie, Asrat Elias Ergena, Eyayaw Ashete Belachew, Asmamaw Emagn Kasahun, Masho Tigabie Teklie, Adeladlew Kassie Netere

**Affiliations:** 1 Department of Clinical Pharmacy, School of Pharmacy, College of Medicine and Health Sciences, University of Gondar, PO. Box: 196, Gondar, Ethiopia; 2 Department of Pharmaceutical Chemistry, School of Pharmacy, College of Medicine and Health Sciences, University of Gondar, Gondar, Ethiopia; 3 Department of Pharmaceutics, School of Pharmacy, College of Medicine and Health Sciences, University of Gondar, Gondar, Ethiopia

**Keywords:** Cardiovascular drugs, Community pharmacy, Dispensing, Prescription-only drugs, Northwest Ethiopia, Simulated patient

## Abstract

**Background:**

Critical actions are required for the proper administration of medications to patients with cardiovascular diseases. However, there has been an increase in irrational use of cardiovascular drugs. The purpose of this study was to determine the extent of non-prescription cardiovascular medicine dispensing practices at community drug retail outlets (CDROs) in Gondar, Northwest Ethiopia.

**Methods:**

A cross-sectional survey and simulated patient-based visits were employed at the CDROs in Gondar City, Northwest Ethiopia between June 1 and July 20, 2022. The cross-sectional component that assessed the self-reported practices used a standardized self-reported questionnaire. A simulated patient (SP) case scenario, using different tracer prescriptions only for cardiovascular medications, allowed for the observation of real-world dispensing procedures. SPSS version 22 was used for the data entry and analysis.

**Results:**

The cross-sectional study approached 76 CDROs, and 71 of them agreed to take part (93.4% response rate). More than half of the respondents (53.5%) were males, with a mean (SD) age of 33.5 ± 9.1 years. Overall, the current self-reported survey showed that 59.2% of the participants provided cardiovascular drugs without a prescription. A total of 213 simulated visits were conducted. Considering all SP scenarios, the percentage of cardiovascular drugs dispensed without a prescription increased to 88.7%. Besides, more than 90% of pharmacists did not demand the SP to have a prescription, did not advise them to visit doctors or clinics, and did not inquire as to whom the medication was required.

**Conclusion:**

A significant proportion of CDROs dispensed cardiovascular medications without a prescription. The findings highlight the disparity between self-reported and actual CDRO practices. Additionally, nearly all of the CDROs approached made it simple to obtain cardiovascular medications. Stakeholders could adherently follow the CDROs’ practices to improve their proper dispensing procedures.

## Background

Professionals who work in community drug retail outlets (CDROs) have a well-recognized duty to dispense medications [1]. Professionals should carefully review the prescription and ask the patient for clarification before dispensing when they are presented [2]. Dispensing is now restricted to the provision of prescription-only medications (POMs), over-the-counter (OTC) drugs, and medical supplies to patients and/or caregivers with valid prescriptions or legal records [3]. In particular, this is especially true in Ethiopia, although it is different practically. In industrialized countries, prescribers and community pharmacists have played distinct roles, with the sole right to prescribe [4].

Because of the rise in the prevalence of cardiovascular diseases, which cause death and are the leading cause of physical and cognitive disability, cardiovascular medications used to prevent and treat these disorders have accounted for many prescriptions in many people. Many recommendations and authorities have since encouraged the routine use of many cardiovascular drugs [5]. However, there is an increasing variation in the prescription for cardiovascular medications based on the patients’ medical problems and treatment settings [5]. Cardiovascular drugs are prescribed to treat diseases of the heart, blood vessels, or circulatory systems such as arrhythmias, blood clots, coronary artery disease, hypertension, high cholesterol, heart failure, and stroke [6], all of which require prescription medications and close monitoring.

CDROs are the most important health sector in underdeveloped countries like Ethiopia because of their wide and easy availability, shorter wait times, and extended working hours [7]. CDROs are classified into different levels based on the types of pharmaceuticals and supplies they are intended to dispense as well as the training of their pharmacists [8–10]. These are drug vendors, stores, and pharmacies with various roles and responsibilities. Pharmacies are now legally allowed to stock and provide any medication, as well as medical supplies. They should be run by licensed pharmacists who are degree holders. Drug stores, on the other hand, have limited offerings and are not designed to stock and supply all medications. For example, holding narcotic and psychotropic medications is prohibited [8–10]. Additionally, all CDROs may require prescriptions for POMs such as antibiotics, cardiovascular drugs, and other controlled medications. However, due to a lack of professional awareness or poor authority control mechanisms, actual practice may deviate from recommendations and standards.

Pharmacists in the CDROs are involved in self-care practices in addition to medication dispensing, especially for patients with cardiovascular problems. They provide patient counseling to manage and prevent cardiovascular diseases [8]. Furthermore, pharmacists are actively involved in consultations when patients or caregivers inquire about a specific prescription or describe symptoms [11]. However, when it comes to POMs dispensing, Africa, particularly Ethiopia, has a high rate of malpractice [12, 13]. To date, nearly every CDRO pharmacist has provided patients and caregivers access to a wide range of medications, including those for chronic medical conditions [8, 12]. Previous research from around the world supported a variety of causes for prescribing malpractice, such as a lack of time and motivation, a lack of awareness and knowledge, and a distance from hospitals and clinics [14–16]. It is critical to regulate the process of dispensing cardiovascular medications to prevent self-medication and ensure proper drug use. The purpose of the prescription may also help patients manage and comprehend their pharmaceutical regimens. To avoid dosing errors, when medications are provided to people of all ages, it can be beneficial to include the patient's pertinent information on the prescription [17].

Despite the fact that community pharmacists have a variety of public health responsibilities in the management and prevention of cardiovascular disorders [8], there is a lack of comprehensive evidence regarding cardiovascular medication dispensing practices in Ethiopia. Although medications are used to combat diseases, they can be seriously harmful if given to the wrong patients [18]. Therefore, the purpose of this community-based study was to assess the extent of non-prescription cardiovascular medication dispensing practices at CDROs in Gondar City, Northwest Ethiopia.

## Methods

### Study design and setting

A two-phase sequential investigation was conducted in Gondar City, Northwest Ethiopia, to determine the extent to which CDROs were dispensing cardiovascular drugs without a prescription. The study was divided into two phases: a self-reported study that used a structured questioner to assess self-reported dispensing practices; and a simulated patient (SP) study that assessed the CDROs’ actual dispensing practices for cardiovascular medications. The first was a self-reported survey and took place between June 1 and 15, 2022. Following that, the SP-based study continued for three weeks with three SP visits. Every SP visit took place within a week of every other visit, which was employed at CDROs that participated in a self-reported survey from July 1 to July 20, 2022.

### Study samples and inclusion criteria

After we identified which CDROs were licensed, we approached every CDRO that was active during the time of data collection. Those who refused to participate and/or were closed during the visit were excluded from the study. One pharmacist from each CDRO took part in the self-reported study component. Consequently, we approached 76 total samples.

### Data collection instruments, procedures and quality control methods

#### Self-administered cross-sectional study component

The data collection instrument was prepared after conducting a literature search, and modifications were made with the local context in mind. The questionnaire was written in English. The self-administered tool was divided into three sections. (I) The first section presented respondents’ socio-demographic information. (II) The second was about the most commonly dispensed cardiovascular medications by CDROs, and (III) the third was expected inquiries, which were made during CDROs’ cardiovascular medication dispensing practice. There were 21 items on a 5-point Likert scale (Never = 1, Rarely = 2, Occasionally = 3, Usually = 4, Always = 5). The items’ internal consistency was deemed satisfactory with a Cronbach alpha score of 0.82.

Prior to the actual data collection period, the questionnaire was pretested in five CDROs; these CDROs were excluded from the final analysis. The goal and procedures of the pretest were explained to each of the five CDROs. After a minor adjustment based on pretest feedback, the self-administered questionnaire was provided to participants who volunteered to take part in this study. Prior to actual data collection, the study participants were briefed on the purpose of the study and asked for their consent to participate. The supervisors explicitly followed the data collection procedures closely. Every day during the data collection period, the data were checked for accuracy, completeness, and cleanness.

#### Simulated patient-based study component

Prior to the actual SP visits, a pretest was given to five CDROs. The pretest was excluded from the final analysis. All five CDROs were informed about the purpose of the pretest and the SP methodology. Following feedback from the pretest and the participating CDROs, the algorithm for the SP and the pharmacist’s dialog was slightly modified. The SP visit was then used to assess community pharmacists’ actual dispensing practices for cardiovascular medications. The dialogue between the SP and the pharmacist was modified using an established SP algorithm [12]. Three graduating pharmacy students who volunteered and had specialized SP roles applied to the SP-based study after a half-day of training on the scenarios and how they communicate. Thus, they became accustomed to and carried out the clinical scenarios provided. The SP pharmacists received effective communication techniques with the working pharmacists. Additionally, the three SP pharmacists’ case presentations were cross-tested to ensure that the data collected were synchronized. To ensure that the information provided to the SPs was consistent, they were instructed not to disclose or request further information unless absolutely necessary. All SP visitors either asked the pharmacist for a specific medication name or presented an empty container of the medication.

#### Simulated patients and scenarios

A 35-year-old male was diagnosed with cardiac disease. In addition, the SP needed to refill the patient’s medications but had no prescription. Furthermore, the patient also had no known comorbidities or drug allergies. However, pharmacists were required to rule out any additional medical conditions, a history of medication use, and whether the patient had visited doctors or clinics. Additionally, on SP visits, pharmacists were expected to obtain valid prescriptions; otherwise, requested medications would not be filled. Furthermore, pharmacists should advise the SP not to take any additional cardiovascular-related medications without a prescription. The pharmacists were also expected to inform the patients that they should be monitored and followed up on a regular basis.

Three visits were made in three weeks, one week each, using three different scenarios. During the study period, the SP visited each CDRO three times to fully understand their actual practices.

In scenario 1, the SP was responsible for asking about two tracer POMs, such as captopril if enalapril was unavailable and atenolol if metoprolol was not available.

Scenario 2 also directed the SP to get information on two tracer POMs, such as propranolol and hydrochlorothiazide (HCT).

In scenario 3, the SP was instructed to ask about three tracer POMs, such as furosemide, spironolactone, and atorvastatin or simvastatin if atorvastatin was unavailable.

The SP data intended to determine the extent of CDROs' non-prescription cardiovascular medication dispensing and counseling practices were filled. The self-reported responses were then compared to the SP-based dispensing practice.

### Data entry and statistical analysis

After being checked for completeness and cleanness, the data from the self-reported survey and SP visits were coded, entered, and analyzed using SPSS Version 22. The normality of the data was examined using the Q–Q plot, histogram, and Shapiro–Wilk test. The study participants’ characteristics were described and presented, using descriptive statistics such as frequency and percentage for categorical variables and mean and standard divisions for continuous variables, tables and figures.

## Results

### Study participants’ socio-demographic characteristics

A total of 71 CDROs out of the 76 that were approached participated in the survey, resulting in a 93% response rate. More than half of the respondents in these facilities (53.5%) were males, with an average age (SD) of 33.5 ± 9.1 years. The respondents had 5.8 ± 4.8 years of work experience on average. More than half of the CDROs included in the study (52.1%) were drug stores. Additionally, more than half of the respondents (52.1%) were employed, and less than two-thirds (62%) had a bachelor’s degree in pharmacy. In terms of employment, about 85% were employed full time (Table 1).

**Table 1:** Socio-demographic characteristics of the respondents to the survey from CDROs in Gondar city, Ethiopia, 2022 (*N* = 71)

Variables	Frequency (%)	Mean (± SD)
Sex		
Male	38 (53.5)	
Female	33 (46.5)	
Age (years)		
18–25	12 (6.9)	33.5 (± 9.1)
26–35	34 (47.9)	
36–45	20 (28.2)	
46–55	2 (2.8)	
> 56	3 (4.2)	
Work experience (years)		
< 1 year	2 (2.8)	5.8 (± 4.8)
1–5 years	36 (2.8)	
> 5 year	33 (46.5)	
Types of CDRO		
Pharmacy	34 (47.9)	
Drug store	37 (52.1)	
Marital status		
Single	25 (35.2)	
Married	45 (63.4)	
Divorced	1 (1.4)	
Level of education		
Diploma	27 (38.0)	
Bacholer's Degree and above	44 (62.0)	
Employment type		
CDRO owner	34 (47.9)	
Employed	37 (52.1)	
Employment status		
Full time	61 (85.9)	
Part-time	10 (14.1)	
Salary per month (Eth. Birr)		
1500–3000	30 (42.3)	4783.1 (± 2757.2)
> 3000	41 (57.7)	
Client visits (per day)		
< 50	64 (90.1)	25.5 ± (15.95)
50–100	7 (9.9)	
Pharmacists’ main role in the CDRO		
Dispenser	69 (97.2)	
Store manager	2 (2.8)	
Know cardiovascular drug class type		
Yes	71 (100)	
No	0 (0)	

### Medication types and dispensing practices without prescriptions

**Fig. 1:**
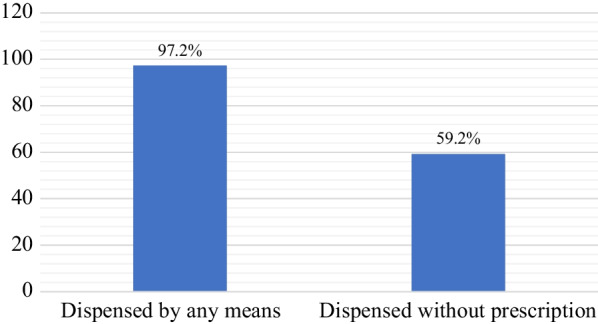
Proportion of participants with dispensed cardiovascular medications in the CDROs

As shown in the graph, the self-reported survey showed that the majority of the participants (97.2%) dispensed cardiovascular medications with or without prescriptions. Almost 60% of participants did not require a prescription to dispense these medications (Fig. 1).

**Fig. 2:**
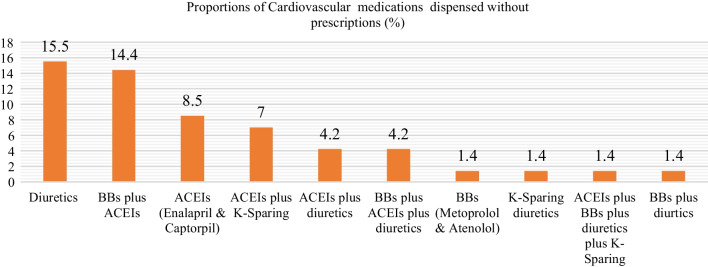
The proportion of cardiovascular medications dispensed in the CDROs of Gondar city

The participants reported that diuretics were commonly dispensed cardiovascular medications (15.5%) at the CDROs, followed by a combination of beta-blockers (BBs) and angiotensin-converting enzyme inhibitors (ACEIs), which was prescribed by 14.1% of participants. Two ACEIs frequently dispensed without a prescription were enalapril and captopril (8.5%) (Fig. 2).

### Queries and interactions from CDROs in response to the medication requests

The overall average score for pharmacists’ interactions and questions in response to requests for cardiovascular medications was 3.8 (± 0.5) out of 5. In response to the advice and instructions provided, the majority of community pharmacists reported that they advised practicing lifestyle and/or non-pharmacologic management. The majority of pharmacists responded that they usually and/or always advised clients to have prescriptions available upon request (78.9%), abstain from alcohol consumption (91.6%), limit salt intake (90.1%), and quit smoking (81.4%), and the mean score was also higher than 4 out of 5. However, a significant proportion of the pharmacists (23.9%) reported that they occasionally dispensed medications without a prescription in response to direct requests (Table 2).

**Table 2:** Queries and interactions in response to the cardiovascular medication enquiries at the CDROs in Gondar city, 2022 (N = 71)

Statements	Never (%)	Rarely (%)	Occasionally (%)	Usually (%)	Always (%)	Mean (± SD)
Dispensing cardiovascular medication by any means	2 (2.8)	6 (8.5)	41 (57.7)	16 (22.5)	6 (8.5)	3.3 (± 0.8)
Dispensing cardiovascular medications without prescriptions	29 (40.5)	20 (28.2)	17 (23.9)	5 (7.1)	0 (0)	2.0 (± 1.0)
Dispensing cardiovascular medications to care provider	16 (22.5)	12 (16.9)	22 (31)	6 (8.5)	15 (21.1)	2.9 (± 1.4)
Encouragement in medication adherence	1 (1.4)	7 (9.9)	19 (26.8)	23 (32.4)	21 (29.6)	3.8 (± 1.0)
Queries on monitoring therapeutic response	1 (1.4)	16 (22.5)	26 (36.6)	19 (26.8)	9 (12.7)	3.3 (± 1.0)
Queries on about drug allergies	6 (8.5)	13 (18.3)	21 (29.6)	15 (21.1)	16 (22.5)	3.3 (± 1.2)
Instruction on drug interaction	6 (8.5)	13 (18.3)	21 (29.6)	15 (21.1)	16 (22.5)	3.5 (± 1.1)
Instruction on frequency and dose	3 (4.2)	10 (14.1)	17 (23.9	28 (39.4)	13 (18.3)	4.3 (± 0.9)
Instructions on duration	3 (4.2)	0 (0)	8 (11.3)	20 (28.2)	40 (56.3)	4.3 (± 1.0)
Instruction on side effects	1 (1.4)	4 (5.6)	15 (21.1)	23 (32.4	28 (39.4)	4.0 (± 1.0)
Queries need for prescription	1 (1.4)	2 (2.8)	12 (16.9)	8 (11.3)	48 (67.6)	4.4 (± 1.0)
Instructions to visit a doctor	1 (1.4)	4 (5.6)	12 (16.9)	26 (36.6)	28 (39.4)	4.1 (± 1.0)
Nonpharmacological advice	6 (8.5)	13 (18.3)	10 (14.1)	17 (23.9)	25 (35.2)	3.6 (± 1.4)
Counseling rate on salt restriction	1 (1.4)	0 (0)	6 (8.5)	16 (22.5)	48 (67.6)	4.6 (± 0.8)
Counseling rate on alcohol restriction	0 (0)	2 (2.8)	4 (5.6)	10 (14.1)	55 (77.5)	4.7 (± 0.7)
Counseling rate to stop smoking	1 (1.4)	3 (4.2)	9 (12.7)	12 (16.9)	46 (64.8)	4.4 (± 1.0)
Advise doing exercise	2 (2.8)	3 (4.2)	11 (15.5	23 (32.4)	32 (45.1)	4.1 (± 1.0)
Advise on weight reduction	1 (1.4)	1 (1.4)	16 (22.5)	22 (31.0)	31 (43.7)	4.1 (± 0.9)
Encouragement rate to have regular checkup	0 (0)	0 (0)	15 (21.1)	22 (31.0)	34 (47.9)	4.3 (± 0.8)
Advise using vegetable diet	2 (2.8)	6 (8.5)	13 (18.3)	23 (32.4	27 (38.0)	3.9 (± 1.1)
Encouragement rate to increase soluble fiber	4 (5.6)	8 (11.3)	21 (29.6)	19 (26.8	19 (26.8)	3.6 (± 1.2)
Overall mean						3.8 (± 0.5)

Never = 1; rarely = 2; sometimes = 3; usually = 4; always = 5

### Extent of cardiovascular medications dispensed to the simulated patient

Out of 213 SP requests, the majority (88.7%) of the pharmacists provided cardiovascular medications in an empty container or upon direct oral request. The vast majority of the pharmacists (89.2%) provided the SP with dosage instructions for the medications they had dispensed. In contrast, only a smaller percentage (1.4%) of the approached pharmacists insisted on the SP presenting the prescription. Furthermore, more than 98% of pharmacists did not advise clients to visit doctors or clinics, did not question the SP for a prescription, did not advise on side effects, smoking, cessation, weight reduction, vegetable diet use, and increased consumption of soluble fiber (Table 3).

**Table 3:** Requests for cardiovascular medication enquiries during the simulated patient visits at the CDROs in Gondar city (*N* = 213)

Statements	Yes	No
Dispenser provided the drugs that you requested	189 (88.7)	24 (11.3)
Dispenser requested SP to have prescriptions?	3 (1.4)	210 (98.6)
Recommended visiting doctors/clinicians before dispensing?	4 (1.9)	209 (98.1)
Dispenser asked for whom the drug is needed?	23 (10.8)	190 (89.2)
Dispenser instructed SP regarding drug–drug interactions?	5 (2.3)	208 (97.7)
Dispenser instructed SP regarding doses of dispensed drugs?	190 (89.2)	23 (10.8)
Dispenser instructed SP regarding duration drugs	60 (28.2)	153 (71.8)
Dispenser instructed SP regarding side effects of the drugs?	2 (0.9)	211 (99.1)
Dispenser recommended visiting doctors/clinicians after dispensing?	28 (13.1)	185 (86.9)
Dispenser recommend any non-pharmacological/lifestyle interventions?	58 (27.2)	155 (72.8)
Advise on restricting the use of salt	42 (19.7)	171 (80.3)
Advise on restrict/stop alcohol drinking	5 (2.3)	208 (97.6)
Advise on stop/cessation smoking cigarettes	3 (1.4)	210 (98.6)
Advise on to exercise	20 (9.4)	193 (90.6)
Advise on weight reduction	1 (0.5)	212 (99.5)
Advise on use vegetable diet	1 (0.5)	212 (99.5)
Advise on increasing the consumption of soluble fiber	0	213 (100)
Advise on regular checkups/monitoring	38 (17.8)	175 (82.2)

**Fig. 3:**
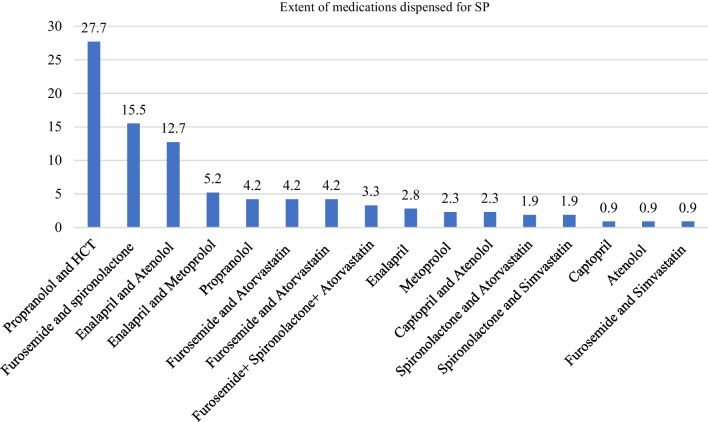
The types and extent of medications provided to the simulated patients

At SP visits, propranolol and HCT were the most commonly dispensed cardiovascular medications (27.7%), followed by furosemide and spironolactone (15.5%) and enalapril and atenolol (12.7%) (Fig. 3).

## Discussion

Despite the fact that numerous studies have been conducted to assess the dispensing practices of various medication classes such as antibiotics, NSAIDs, opioid analgesics, naloxone, and so on, there is a paucity of evidence regarding the dispensing practice of cardiovascular medications in CDROs in Northwest Ethiopia, particularly at the study setting of Gondar City. Consequently, the current dual-phase study attempted to assess the dispensing practices in CDROs through both a self-reporting interview and the presentation of an SP case scenario. Community pharmacies can play an important role in accessing medications that are used to treat several medical disorders. However, because patients can easily access their preferred medications, the non-prescription distribution of POMs such as cardiovascular medications increase self-medication. Self-medication has a few advantages, but it also has a number of well-known risks and negative effects [19]. Self-medication has a very high risk, in particularly in individuals with cardiovascular diseases. Furthermore, since its inception, drug dispensing has generated a variety of ethical and legal problems, particularly in CDROs. Patients are using CDROs as a shortcut to access medications for their disease conditions without visiting doctors in order to avoid additional costs of money and time. Patients, however, compromise the system of rational drug use, which causes a number of problems. Therefore, it is critical to improve pharmacists’ POM dispensing practices to reduce medication dispensing malpractices that could have significant outcomes. Thus, determining the extent of dispensing practice is important. The current study also aimed to evaluate the community pharmacists’ self-reported and actual dispensing practices for cardiovascular medications.

The self-reported cross-sectional study in this investigation showed that nearly all the pharmacists dispensed cardiovascular medications with or without prescriptions. Additionally, a significant proportion of responders dispensed medications without a prescription. In contrast to the self-reported findings, a much higher proportion of pharmacists dispensed prescription cardiovascular medications to SPs with only an oral request or an empty container. The approached pharmacist at the CDROs did not frequently require the SP to have a prescription or consult a doctor before taking medications. Surprisingly, the majority of the pharmacists provided the requested medications without seeking justification. The findings of the SP-based study were highly correlated with those of a simulated study conducted in Addis Ababa, Ethiopia, which assessed the practice of dispensing some selected drugs without a prescription and found that 89.6% of captopril was dispensed without a prescription [12]. These findings prove that there is a significant gap between the self-reported survey and the SP scenario visits. This numerous and clear lack of correlation can be attributed to issues of societal acceptance. This was also partly justified by the fact that community pharmacies are run as businesses under the loose supervision of the relevant authorities. This implies that almost all cardiovascular medications are given to patients on their own without the consent or prescription of a doctor. Another SP study conducted in Saudi Arabia showed that the dispensing practice of antihypertensive medications, which are considered cardiovascular drug classes in the current study, was more common without a prescription [20]. Overall, our findings shared several similarities with previous studies. However, when compared to the previous study’s findings on dispensing malpractices, the current finding is significantly higher [21]. In addition, in contrast to the current finding, a study conducted in the Gaza strip revealed that the majority of pharmacists were hesitant to dispense drugs for cardiovascular diseases and diabetes mellitus [22]. The discrepancy may be due to differences in authorized body monitoring and controlling mechanisms, as well as pharmacists’ knowledge, attitude, and practices regarding patient counseling and dispensing activities for those patients with chronic diseases. Therefore, the findings might indicate that regulatory organizations may need to monitor and follow up on services. Training on POM dispensing procedures and rational medication use, particularly for cardiovascular medications, should also motivate community pharmacists.

The mean scores of the provided queries and interactions that pharmacists carried out in response to requests for cardiovascular medication requests were notably higher than the average levels in the self-reported study. Lifestyle changes or non-pharmacologic management of cardiovascular problems are frequently recommended and given advice. For this, pharmacists usually advise patients to avoid alcohol, limit salt intake, quit smoking, and insist that medications be available upon prescription request. Additionally, about one-third of pharmacists provided medications to caregivers, and only about one-third of respondents reported that they could monitor therapeutic outcomes and inquire about drug allergies and interactions. This is in line with the previous study [8]. In the SP-based study counter checkup, the pharmacists preferred to instruct the dosage information for the medications that were already dispensed. However, a much smaller number of them also provided dosage regimen duration instructions and recommended non-pharmacological interventions to prevent and treat cardiovascular disorders. Likewise, during SP visits, a significantly higher percentage of pharmacists did not advise the inquires to visit doctors or clinics, did not inquire about the need for medications, and did not provide instructions regarding side effects, quitting smoking, losing weight, using a vegetable-based diet, and increasing soluble fiber intake. As this dual method approach study practically confirmed, the actual and theoretical concepts of dispensing practices are very different, and dispensing malpractices are higher. Although it was expected that pharmacists would adhere to theoretical and scientific pharmaceutical dispensing standards, the reality of this SP case scenario proved that this was completely different from what was assumed to be. This malpractice is also significantly higher than the findings from similar studies conducted in Addis Ababa, Ethiopia [12, 21] and India [23]. Community pharmacists’ reluctance to comply with the dispensing rules and regulations, which they consider CDROs as a business sector, could be one reason for such a higher malpractice rate. To achieve better results in preventing the misuse of such pharmaceuticals, academic institutions, communities, authorized bodies, and pharmacists themselves should all work together.

The self-reported results of the most frequently dispensed medications revealed that diuretics were the most frequently dispensed medication without a prescription, followed by ACEIs and beta-blocker combinations. In the SP report, the most frequently dispensed cardiovascular medications without a prescription were a combination of propranolol and HCT, furosemide and spironolactone, and enalapril and atenolol. These findings revealed that diuretics, beta-blockers, and ACEIs are the most commonly used drugs without a doctor’s prescription or patient monitoring. These medication groups, however, require clinical and laboratory-based monitoring for improved treatment outcomes in patients with cardiovascular problems, including fluid status, electrolyte levels, renal function, metabolic abnormalities, and heart dynamics. The findings justify that misuse of these types of medications is common, though it is also controllable. Therefore, to tackle misuse of POMs, especially cardiovascular medications, it is critical that local and regional guidelines be developed, as well as strict monitoring and control from the relevant authorities.

In terms of the non-pharmacological advice given to the clients, the findings of the current study differed favorably from those of previous studies conducted in Ethiopia [12] and India [23]. Furthermore, even if the difference was not statistically significant, more pharmacists in the current study were engaged in non-pharmacological advising practice than in previous studies conducted in Ethiopia and India. This improvement could be attributed to the current study’s focus on cardiovascular diseases, which must be managed with non-pharmacological lifestyle changes. In fact, this strategy serves as the first line of management for the majority of diseases. Therefore, even though the non-pharmacological counseling practices have increased as planned, the overall result regarding the dispensing practices of cardiovascular medications remains below what is recommended.

In general, both self-administered and SP-based studies show a much higher extent and dispensing rate of cardiovascular medications without a prescription, with the SP case scenario-based study significantly higher. Therefore, regulatory bodies such as the Ethiopian Food and Drug Authority (EFDA) would play a significant role in controlling and monitoring processes. Additionally, new policies, laws, and procedures may be implemented to reduce medication misuse and ensure patient safety. Incorporating pharmacist interventions could improve patient safety and reduce the risk of dispensing cardiovascular medications without a prescription.

### Study limitations

There are some limitations to the current study. First, because we used a small sample size, our findings may not be generalizable to all CDROs in the country. The findings regarding the dispensing practices of cardiovascular medications without a prescription may be over or under reported in the self-reported study because it is dependent on the respondents’ honesty and faith. Therefore, these findings should be interpreted cautiously. Furthermore, larger sample population research may be recommended. Despite the limitations mentioned above, the current study highlighted the extent of current practice in the dispensing of cardiovascular medications in CDROs, and we hope it will fill the existing literature gap in the study area.

## Conclusion

According to the findings of this study, the rate of dispensing cardiovascular medications without a prescription is high. The findings also highlighted the existing gap between self-reported and actual CDRO practices. Furthermore, cardiovascular medications were easily obtained from almost all of the CDROs approached. Therefore, developing local and regional guidelines as well as close monitoring and control from authority bodies are critical for proper medication dispensing practices. Additionally, responsible stakeholders should provide regular training to pharmacists on proper POM dispensing practices.

## Data Availability

The datasets generated and/or analyzed during the current study are available from the corresponding author on reasonable request.

## Abbreviations

CDROs: Community drug retail outlets
EFDA: Ethiopia Food and Drug Authority
POMs: Prescription-only medications
